# Design and application of oral colon administration system

**DOI:** 10.1080/14756366.2019.1655406

**Published:** 2019-10-03

**Authors:** Hao Cheng, Shiyu Huang, Gangliang Huang

**Affiliations:** Chongqing Key Laboratory of Green Synthesis and Application, Active Carbohydrate Research Institute, Chongqing Normal University, Chongqing, China

**Keywords:** Oral colon administration system, design, application

## Abstract

Oral colon administration system has become a new method to treat intestinal diseases. The implementation of colon drug delivery system is restricted by many aspects, including physical and chemical properties, drug delivery mode, gastrointestinal physiological factors, and so on. Delivery methods to overcome these challenges revolve around the mechanisms of drug delivery, including the use of rational dosage forms to avoid the complex pH environment, and the prevention of drug release and absorption in the upper digestive tract.

## 1. Introduction

Colon is an important digestive organ of organism, and also the main site for microbial flora colonization. The microbial content in colonic contents can reach 10^9^–10^11^/g, accounting for 85–90% of the total microbial flora in intestinal microecosystem. Microbial flora constructs a large external environment of microecosystem in colon^1–3^. Colonic colonies play an important role in digestion by decomposing macromolecule polysaccharides which are difficult to digest. Colonic microorganisms also play an important role in regulating intestinal and immune functions^4^. *C. Bacillus* in intestinal flora can synthesize the important intermediate product indole propionic acid (IPA) by decomposing tryptophan, strengthen the intestinal wall and enhance the absorption of intestinal substances^5^. The experimental mice had autoimmune diseases by knocking out the PD-1 receptor and taking intestinal flora as the research object^6^. In recent years, due to the abuse of antibiotics and other issues, research on colonic and intestinal flora has attracted much attention, such as diarrhea related to antibiotic abuse^7–9^, colonic inflammatory bowel disease (IBD)^10–12^, intestinal flora of AIDS patients contains more *Escherichia coli* and *Pseudomonas aeruginosa*, and the content of Lactobacillus has significantly decreased^13^^,^^14^.

At present, oral medicines are the most commonly used way to treat colon diseases, but the delivery of polypeptide drugs is very vulnerable to gastric acid or secretory protease degradation and inactivation in the digestive tract. At the same time, the drug is absorbed into the blood by the body before reaching the colon, which makes it difficult to increase the drug concentration at the focus and reduce the therapeutic effect. Therefore, the improvement of oral pharmaceuticals has attracted wide attention.

## 2. Advantages and development of oral colon-targeted drug delivery system

In order to overcome the shortcomings of oral drug delivery system in the treatment of intestinal diseases, a new oral drug delivery system, namely oral colon-targeted drug delivery system (OCTDDS), has been introduced into the research field. By means of pharmaceutics such as drug modification and coating, the system avoids direct absorption of drugs in the anterior gastrointestinal tract after oral administration, and interprets drugs in the blind junction to achieve local therapeutic effect. As a fourth-generation drug formulation, the system can provide technical support for oral polypeptide drugs, which can prevent the degradation of proteinase in gastrointestinal tract and inactivate them^15^^,^^16^. At the same time, it can improve the concentration of targeted drug by improving the targeting site orientation, enhance the therapeutic effect, and reduce drug use and toxic side effects. Therefore, the key issues of the system are targeting drug delivery mechanism, selecting drug carriers, and modifying carrier materials.

At present, in terms of drug delivery mechanism, the system mainly achieves targeted drug delivery through the physiological characteristics of colonic segments and microbial releasing enzymes colonized in colonic sites, such as: (1) The colon pH ranges from 6.5 to 7.5, which is mainly affected by food structure and body condition. (2) The colonic segment is rich in microbial flora that can secrete macromolecule material degradation enzymes. (3) The peristalsis rate of colon segment is slow, and the content stays in colon for a long time. (4) The colon absorbs a large amount of water, which leads to the solidification of the contents and the increase of intraluminal pressure. According to these physiological characteristics, traditional drug delivery systems based on colonic physiological characteristics, such as pH-dependent^17^, time-dependent^18^, and pressure-controlled^19^, have been proposed. In addition, passive targeting drug delivery systems initiated by microbial flora, such as enzyme-triggered^20^, prodrug delivery systems^21^, enzyme-degradable polymer-coated drug delivery systems^22^, and complex colon targeting drug delivery systems^23^, which combine several delivery systems, are used. In the field of drug carrier materials, chemical modification of carrier materials or carrier materials with strong sensitivity and specificity, stable drug release process, and nontoxic side effects is currently a research hotspot in this field.

## 3. Traditional drug delivery system based on colon physiological characteristics

The pH of gastrointestinal tract in animals increases continuously from stomach to small intestine and then to large intestine. During the period of no food digestion, the pH of gastric juice is 1–2, the pH of jejunum and ileum is about 6.5 and 7.5. After the cecum segment, the pH of colon first decreases and then increases, the pH of colon increases to 5.4, and the pH of transverse colon and descending colon increases from 6.6 to 7.0. According to the difference of pH in different parts of gastrointestinal tract, pHOCTDDS was designed. According to the molecular structure of target drug, chemical modification or pH-sensitive material was carried out to coat the drug. Drug release in stomach and small intestine was controlled under acidic conditions. When pH was about 7.0, chemical bond breakage or coating material disintegration occurred between carrier material and drug in colon segment, released drugs, and ultimately achieved colon targeting effect^24^^,^^25^.

At present, the most commonly used carrier material for pH-dependent drug delivery systems is acrylate copolymer (Eudragit), which can obtain different pH sensitivity by changing R group^26^. For example, pH-dependent enteric coated Eudragit S100 based on nanosuspension has the characteristics of small particle size and large surface area, which can effectively improve drug permeability and drug dissolution rate.

Baicalin was used as the target drug to prepare nanosuspension. Eudragit S100 was used to coat the drug. The drug was dissolved and disintegrated at pH >7 to release the drug. In order to solve the problems of brittleness of Eudragit S100 and high glass transition temperature of the coating, plasticizer TEC and talcum powder were added to reduce the adhesion between nanoparticles. It was found that the drug was used in artificial gastric juice under acidic conditions for 2 h and in small intestinal juice for 4 h. The dissolution rate of the drug was low, while the drug was released in a large amount in the slightly alkaline artificial colon solution, which proved that it had a good colon targeting effect^27^^,^^28^.

Synthetic polymer coating materials affect the immunity of organism, and polysaccharides are widely used in the preparation of drug coating. Alginate and carboxymethyl chitosan were used to prepare a hydrogel material with network interpenetration at molecular level. When pH = 1.2, there was almost no drug release. When pH reached 7.4, the drug release increased, which made the drug specifically act on the target site^29^. When β-cyclodextrin and retetracete were prepared into tablets, the drug was safely delivered through the gastric and small intestinal segments and finally released in the colon. Compared with oral retetracete tablets, the cumulative distribution of β-cyclodextrin and retetracete in the colon tissue was increased by 6.3 times^30^.

**Figure 1.**
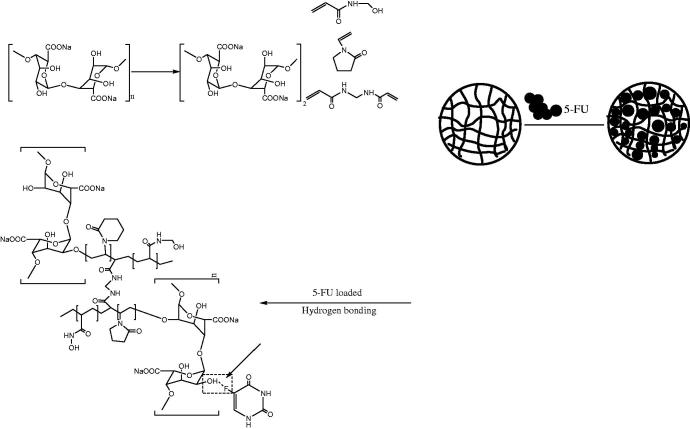
Preparation and mechanism of 5-FU loaded on NaAlg-*g*-p (NVP-*co*-NHMAA).

In recent years, researchers have improved the morphology of carrier materials at the molecular level through advanced instruments to improve the drug release effect. In order to overcome the poor strength and stability of NaAlg hydrogels, pH-sensitive and porous NaAlg-g-P (NVP-co-NHMAA) hydrogels were prepared by ultrasound-assisted free radical graft copolymerization to reduce their release in acidic environment (Figure 1)^31^.

The preparation method of pHOCTDDS is simple, low cost, and easy to operate. However, pathological changes of gastrointestinal tract or poor dietary structure in animals lead to the fluctuation of gastrointestinal pH, which leads to premature disintegration of drug-coated pH-dependent polymer materials and affects the effect of drug treatment. Therefore, it is limited in clinical use^32^.

Time-dependent colon-targeted drug delivery system guides drug release according to the time when food arrives at the focus after oral administration. In general, gastric emptying time is 15–180 min, while chyme retention time is 3–4 h in the small intestine, and time-dependent delay is usually set to 5–6 h^33^. At present, the drug is coated with insoluble coating materials which are difficult to decompose, and the drug release time is controlled by controlling the proportion and dosage of coating materials.

PEG6000 and PEG4000 are commonly used to coat the target drug. For example, the extraction of oridonin from plants limits its clinical application because of its poor water solubility. The oridonin dropping pills were prepared by pressing oridonin powder tablets using PEG6000 and PEG4000 insoluble materials, and non-permeable cysts were prepared by taking certain ethyl cellulose. Finally, the release delay of oridonin reached 5–6 h at the colon action site^34^.

With the development of technology, chemical modification of mature time-delay materials to obtain drug delivery systems with good drug release effect has become a research hotspot. For example, mesalazine for ulcerative colitis was coated with hydroxypropyl cellulose and microcrystalline cellulose, and then coated with isolation layer and Eudragit-RS30D time-lag layer by organic acid (tartaric acid) induction to prepare organic acid-induced TDCT. It was found that organic acid-induced TDCT had a special slow-release effect in conventional time-lag release, while the uninduced coated TDCT had a sudden release effect^35^.

In order to find cheap and environmentally friendly carrier materials, researchers are paying more and more attention to polysaccharides widely existing in nature. For example, sodium hydroxyacetate and diclofenac were prepared into tablet pills and coated with Eudragit RSPO, a traditional Eudragit polymer derivative, to obtain a time-delay drug delivery system. *In vitro* experiments showed that the drug delivery system containing 5% Eudragit RSPO could keep drug release within 5 h with good delay effect^36^.

However, the system mainly controls drug release through the thickness of the coating material. The cost is high and the difference between the organisms is large. The retention time of the intake in different intestinal segments of the animal body varies greatly, and the pharmacodynamics and bioavailability of the drug are limited. At present, the system is mainly optimized by combining with other systems^37^.

Pressure-controlled drug delivery system uses peristaltic waves generated by the characteristics and regular peristalsis of the colonic solidification content to instantly increase the internal pressure of the colonic segment, so that the encapsulated carrier material disintegrates and releases the drug. There are peristaltic waves in the body's gastrointestinal tract, but a large amount of small intestinal fluid in the small intestinal segment can effectively buffer the intraluminal pressure, and the intraluminal pressure of intestinal contents decreases. However, the colon absorbs a large amount of liquid, and the contents solidify, the intracavitary pressure is increased in the colon, the carrier material is cracked, and the drug is released to achieve colon targeting effect^38^.

At present, polymer materials which are not affected by pH and time are mostly used in pressure controlled release systems. Taking enteric-coated (PCDC) as an example, the small intestinal fluid buffer makes the small intestinal cavity pressure insufficient, and the coating can not rupture. When reaching the colon, combined with the effect of colonic solidification, the pressure of PCDC drugs in the colon cavity increases and the content of drugs is released. Capsule technology is currently more commonly used. For example, pressure controlled release system-PCDC (pressure controlled release capsule) could be used to coat caffeine, the target drug was dissolved in water-soluble or fat-soluble matrices, such as polyethylene glycol or synthetic fatty acids, and it was coated with enteric-soluble capsules. The outermost layer was coated with ethyl cellulose (EC), which was insoluble in water and has better sustained-release effect^39^. By controlling the thickness of ethyl cellulose material and choosing to release drugs in different parts of colon, the drug release was less than 25% in artificial gastric juice for 2 h and artificial intestinal juice for 4 h. According to the results of *T*_max_ (5.67 ± 1.21) h and MRT (16.80 ± 1.74) h, caffeine was released and absorbed in the colon.

At present, pressure colon targeting drug delivery system materials are usually combined with coating technology-capsules to control drug release. The study on the physiological function of intra-colonic pressure is still in its infancy and has great development space^40^.

## 4. Passive targeted drug delivery system initiated by microbial flora

The colon targeting system of prodrugs refers to the inactive prodrugs formed by chemical bonding between active drug components and polymer carrier materials. Polymer carrier materials are only sensitive to the release of bacterial enzymes from certain bacteria in the colon segment. After reaching the specific colon flora position, the polymer carrier materials are degraded by specific bacterial enzymes and release the active ingredients at the preset sites. This can increase the drug concentration at the site to improve the efficacy^41^.

At present, azo, glycosides, and dextrans are commonly used as carriers in colon-targeted drug delivery systems, among which azo carriers are relatively mature. 5-Aminosalicylic acid (5-ASA) can be used to treat colitis. Sulfadiazine was used as a carrier material to modify the prodrug with 5-ASA azo bond. As a targeted drug for colon, *in vitro* experiments showed that the prodrug could not be released in the gastrointestinal tract due to the lack of azo-degrading enzyme. After reaching the colon segment, the prodrug was degraded by the azo reductase released by colonic flora, and the azo bond was cleaved to form amino group. The amino group was hydrolyzed by ester bond. Finally, the active 5-ASA, which can treat colitis, was released and the drug targeting was achieved^42^. Sulfadiazine has some toxic and side effects, while butyrate has been exploited for its advantages of fast metabolism, low bioavailability, and short half-life. Butyrate and 5-ASA were linked by azo bond, and nontoxic glycerol was used in the synthesis process. Accordingly, the toxicity and side effects of the prodrug delivery system could be eliminated and the advantage of colon targeting could be maintained. In addition, modification of precursor materials with physiological functions has become a new research method, such as inflammatory colitis, because mucin in colonic mucus is easily acidified by chyme, which damages colonic mucus. Acidification sites are mainly targeted at oligosaccharide side chains of mucin. Aminosaccharides improve colonic mucus toughness by protecting mucin structure and forming a protective layer on the surface of colonic mucosa against acidification. Mycophenolic acid (MPA) is a prodrug system composed of covalent amide bonds and glucosamine or glucan as carrier materials. It not only improves the hydrophilicity of drugs, reduces the release of drugs in the upper gastrointestinal tract (GIT), but also releases MPA and aminoglycan precursors under the secretion of specific enzymes (*N*-acylamidase) by colonic microflora. Radiation protects colonic mucosa and optimizes the therapeutic effect of IBD^43^.

At present, the key problem is to select the right drug carrier material for POCTDDS. Because the cost of prodrug delivery system is too high and most precursor materials have some side effects, clinical application is limited^44^.

There are a large number of microbial flora in the colon of healthy animals, secreting and degrading bacterial enzymes such as β-glucosidase, β-glucosidase, cellulase, nitroreductase, azo reductase, α-dehydroxylase, cholesterol dehydrogenase, and so on^45^. The drug delivery system is called bacteria triggered BtOCTDDS (Enzyme-triggered colon targeted drug delivery system), which uses specific bacterial enzyme-sensitive macromolecule materials as drug carriers and modifies drugs through coating or capsule technology to release drugs in the colon. It has the advantages of high biocompatibility, safety, nontoxic side effects, and strong targeting^46^.

Using xanthan gum and guar gum coated with metronidazole file, stomach and small intestine fluid were simulated with 0.1 mol/l hydrochloric acid and pH 7.4 phosphate buffer, and colon fluid was simulated with 4% W/V cecum content pH 6.8 phosphate buffer. The drug release rate *in vitro* was only 12%–33% in stomach and small intestine in the first 5 h, reaching the colon segment and drug release in large quantities^47^.

Compared with traditional drugs, drug delivery systems of carrier proteins and polypeptides have developed rapidly. With bovine serum albumin (BSA) as a model protein drug model, the chitosan-loaded nanoparticles were first prepared by ionic gel method. Using alginate as the shell layer with slow release effect, coaxial nanoparticles were prepared by coaxial electrospinning. It was found that the two level structure of encapsulated BSA hardly changed, and no release was found in the stomach and small intestine. The ileocecal 75% BSA was released in the simulated colon fluid^48^. The main carrier materials of BtOCTDDS are azo and polysaccharide polymers, and the polysaccharide carrier materials are water-soluble polymers.

In addition to using polysaccharide polymer as carrier material, the carrier material can also be modified by adding metal ions, polysaccharides, lipids, and other substances^49^. Ketoprophene (KTF) was used as the drug model, pectin was used as the carrier material, chitosan and lecithin were added to form the composite mixed gel ball, and metal ions (Ca^2+^ and Zn^2+^) were added to study the modification of carrier material. Chitosan and lecithin could increase the encapsulation efficiency of pectin carrier (from 57.59% to 77.63%). Zinc pectin system released 10% in simulated small intestinal fluid (SIF) and 83.21% in simulated colon fluid (SCF), which proved that metal ions can improve the performance of carrier materials.

BtOCTDDS is a passive targeting mechanism for the degradation of polysaccharide carrier materials by bacterial enzymes released from microbial flora. It has the advantages of high bioselectivity and nontoxic side effects. The key to the use of enzyme-triggered colon-targeted drug delivery system is to select the carrier polymer materials correctly and obtain high-quality carrier materials through reasonable modification and modification of other polymer materials^50^. At present, the use of other molecular materials to modify carrier materials remains to be further studied.

## 5. The complex OCTDDS (COCTDDS)

Integrated colon targeting drug delivery system is a colon targeting drug delivery system constructed by combining two or more mechanisms of colon targeting^51^. There are some drawbacks in single-factor drug delivery system, such as the pH-dependent type is affected by the physiological state of the animal body and the types of food; the time-controlled targeting system is affected by the individual differences between the animal body; the target drug is modified by the precursor targeting drug delivery system, which is susceptible to interference from external factors. In order to enhance the accuracy and stability of drug delivery system, the combination of multiple oral colon targeting systems has become a research hotspot^44^. The main reagents of COCTDDS were shown in Table 1.

**Table 1.** Main types and representative drugs of COCTDDS.

Modifying material	Carried drug	Representative type
Alginate gel/Chitosan	Emodin	Alginate gel/chitosan composite particles^52^
Eudragit/Chitosan	Indomethacin	Eudragit-chitosan membrane colon targeted mini dispersible tablets
Ethyl cellulose (EC)/ Eudragit FS30D	Curcumin	Curcumin pills
Eudragit S/Eudragit RS	Indomethacin	Electrospinning nanofibers indomethacin

COCTDDS mainly includes pH and time-dependent colon-targeted drug delivery system, pH-flora/enzyme-triggered colon-targeted drug delivery system and pH-dependent/bioadhesive colon-based drug delivery system. Curcumin was coated by centrifugal granulation and EC and Eudragit FS30D were coated according to pH and time-dependent colon targeting drug delivery system^53^. The delayed inner coating solution was composed of 3.0% EC, 0.6% diethyl phthalate, and 25% ethanol; the weight of the coating is controlled by 2.0%. The outer coating solution based on pH was composed of Eudragit FS30D, 40% talc powder, and 3.0% triethyl citrate, and the weight of the coating was controlled by 4.0%. The results showed that the cumulative release rate of pellets from artificial gastric juice was less than 15%, and the cumulative release rate of pellets from artificial intestinal juice was more than 85% in 5 h, which had obvious colonic targeting characteristics.

The material modification is also one of the means to improve the targeting effect. For example, Eudragit S and Eudragit RS were coated with anti-inflammatory drugs in the form of nanofibers by coaxial electrospinning. Nanofibers had the advantages of porous and large body surface area, which could enhance the drug release effect^54^. In addition, using lactic acid glycolic acid copolymer (PLGA) and pH-sensitive methacrylic acid copolymer as carrier materials, nano-modified carrier materials were used to analyze the therapeutic effect and drug release effect of budesonide nanospheres in mice with colitis. It was found that budesonide nanospheres were enriched in the area of colon lesions in mice, which proved that budesonide nanospheres had a good colon targeting effect *in vivo*^55^.

In the study of pH-enzyme triggered colon-targeted drug delivery system, dextran was used as matrix material to prepare cross-linked microspheres C (Dex-g-PSSS) by graft polymerization and inverse emulsification cross-linking technology^56^^,^^57^. Under the condition of pH = 2, the gelled microspheres with double control of enzymes and pH had strong adsorptive power to 5-fluorouracil, and hardly release the drug. However, under the condition of pH 7.2 in small intestinal fluid with glucanase, the drug was suddenly released in colon region. In addition, pH-dependent materials such as Eudragit and chitosan, the trigger materials of polysaccharide microbial enzymes, have also been developed and utilized. For example, solid nanoparticles made of enzyme-triggered chitosan indomethacin were prepared and Eudragit resin was used as a pH-dependent enteric-coated material to prepare the double-layer coated mini-tablet. Finally, the *in vivo* fluorescence imaging technology in animal experiments proved that this system could target the colon^58^.

Therefore, the integrated colon targeting system has a multi-factor regulatory mechanism, which can improve the effect and accuracy of colon drug targeting. This method has become the most valuable drug delivery method at present. By controlling the thickness of the coating material and using various sensitive materials to protect drugs in many directions, the accuracy of oral colon targeting drug delivery system can be improved, and the individual differences of the body can be weakened^59^.

## 6. Conclusion

In recent years, oral colon targeting drug delivery has become a breakthrough to solve the problem of intestinal flora disorders. OCTDDS system guarantees the release of traditional drugs in colon, increases the concentration of drugs in focus sites, reduces the toxicity and side effects of drugs, and also protects polypeptide and protein drugs from degradation of gastrointestinal protease and avoids pain caused by injection therapy. OCTDDS system still has many shortcomings. In clinical use of pHOCTDDS and TdOCTDDS, individual differences and different health status of the body will lead to the early or delayed release of drugs. The safety of carrier materials needs to be improved. POCTDDS has slight toxic side effects on ligands released from precursor materials after drug release. Although BtOCTDDS and PCOCTDDS use polysaccharide polymer materials, which are safe and nontoxic, there are many problems in the selection of polymer materials and drug mixing. COCTDDS can make up for the shortcomings of different drug delivery systems and reduce the interference of internal and external environment by using different subsystems, but the technology needs to be improved.

In conclusion, the systematic study of OCTDDS will provide a more effective way of drug delivery for the treatment of intestinal microflora disorder, intestinal inflammation and colon-targeted drug release. At the same time, it will provide a driving force for the research and development of biomaterials and a new design method for targeting delivery system.
